# Hydrophobicity of Rare Earth Oxides: Contrasting Perspectives and Emerging Insights

**DOI:** 10.1021/acs.cgd.5c01025

**Published:** 2025-09-15

**Authors:** Jayna K. Patel, Ivan P. Parkin, Claire J. Carmalt

**Affiliations:** Materials Chemistry Centre, Department of Chemistry, 4919 University College London, London WC1H 0AJ, U.K.

## Abstract

The wettability of rare earth oxides (REOs) including the lanthanide series, scandium, and yttrium has become a subject of increasing interest and debate. While many studies report hydrophobic behavior with high water contact angles, emerging evidence indicates that pristine REO surfaces are intrinsically hydrophilic, and that hydrophobicity arises primarily from extrinsic surface contamination by volatile organic compounds. This perspective examines the contrasting viewpoints on REO surface wettability, integrating insights from surface structure, electronic configuration, and environmental interactions. We evaluate how factors such as crystal orientation, defect density, and chemical bonding influence water–surface interactions and contribute to the dynamic nature of REO hydrophobicity. Particular attention is given to recent findings that link hydrocarbon adsorption to changes in surface energy, and to how synthesis and surface modification techniques can tailor wettability. This evolving understanding has broad implications for applications in catalysis, biomedicine, coatings, and energy. We propose that future research should focus on isolating intrinsic surface properties from environmental effects to achieve precise control over REO wettability.

## Graphical abstract

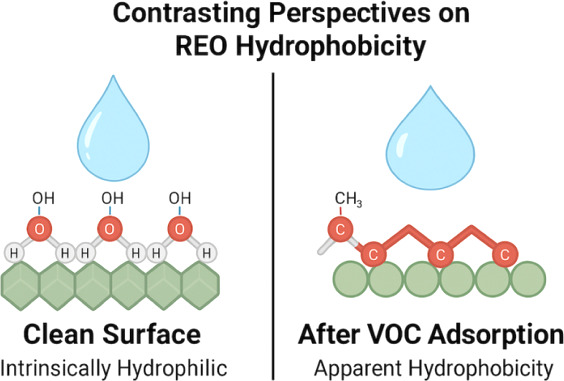

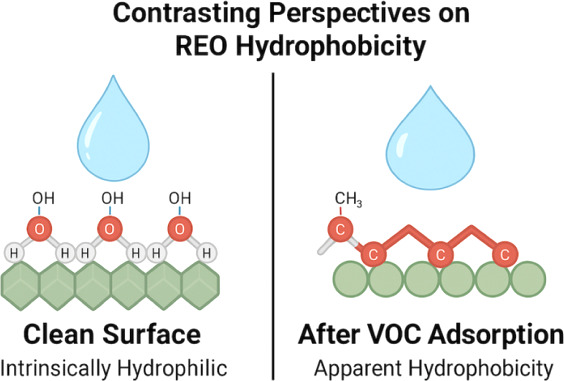

## 1 Introduction

Rare earth metal oxides, encompassing the lanthanide series (including scandium and yttrium), continue to captivate researchers worldwide due to their exceptional chemical properties. These oxides play an essential role in diverse applications, including catalysis,^1–7^ semiconductors,^8–10^ batteries,^11–15^ ceramics,^16–18^ and glass.^19,20^ Although their overall abundance is relatively high, their rarity arises from the dispersed distribution of these elements across geological formations. Owing to their name, these elements occur in rare minerals in the Urals, Scandinavia, United States, Greenland and Brazil as silicates in granite rocks. Although their actual abundance is comparable to that of elements like copper, their limited accessibility has led to their alias as rare earths. Examples of rare earths in their natural state include cerite, allanite and xenotime where each commonly contains several earths. Today, rare earth oxides (REOs) underpin cutting edge technologies, underlining their growing commercial significance as a highly valuable group of trace elements.

The electronic configuration of the lanthanides – involving the progressive filling of the 4f orbitals – provides REOs with valuable magnetic, catalytic, and luminescent properties for industrial use. The characteristic filling of the inner 4f orbitals in rare Earth elements (REEs) provides minimal shielding of the outer electrons from nuclear charge, facilitating the stabilization of their predominantly trivalent oxidation state.^21,22^ Thus, with the exception of tetravalent cerium, most REOs are trivalent and display consistent physical and chemical traits, including thermal stability, magnetism, luminescence, and distinctive wetting behaviors.^23,24^ These atomic and surface properties will be discussed in detail bid. Despite their inherent properties suggesting predictable wettability, recent literature reveals conflicting observations regarding their interaction with water. This review aims to examine these contrasting perspectives.

Hydrophobicity refers to the physical property of a material in its ability to repel water by a contact angle of 90° or higher.^25^ Consequently, surfaces which exhibit a water contact angle (WCA) of 150° or higher are classified as superhydrophobic.^26^ Fueled by a high surface tension of water and low surface free energy, as demonstrated in Figure 1, these surfaces offer a means to anticorrosion and self-cleaning materials by preventing the absorption of water, attracting interest among materials scientists. Materials with a higher surface energy are typically hydrophilic and retain a static water contact angle <90°.^27^ This threshold for hydrophobicity derives from Young’s model, where θ_0_ is the Young’s CA, γ_SG_, γ_SL_, and γ_LG_ represent the surface tensions between solid–air, solid–liquid and liquid–air interfaces, respectively.^28^ REOs with hydrophobic properties present a vast range of applications due to their unique surface properties, chemical stability and versatility. Superhydrophobic surfaces have also gained significant attention for their application in self-cleaning and anticorrosion coatings.^29–31^

**Figure 1:**
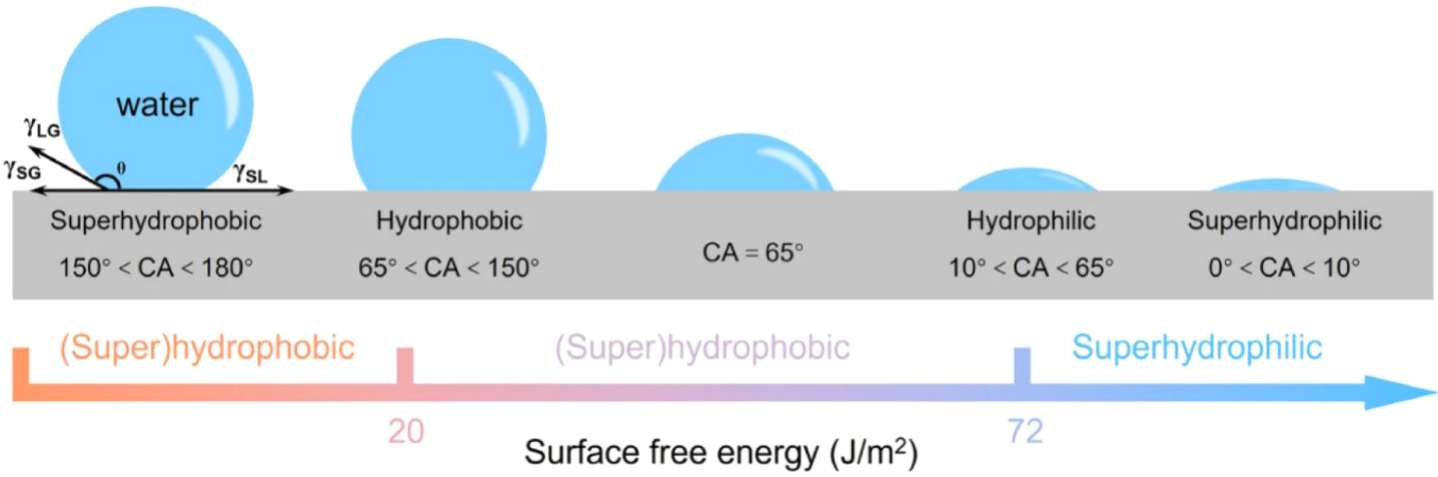
Wettability of surfaces defined by the WCA observed and inherent surface free energy. Adapted with permission from ref 26. Copyright 2025 American Chemical Society.

Coatings synthesized using REOs, such as cerium oxide (CeO_2_) and lanthanum oxide (La_2_O_3_), are particularly effective in creating hydrophobic surfaces. Water contact angles (WCAs) of up to 159° and 157° have been achieved using CeO_2_ and La_2_O_3_, respectively, through various synthesis methods.^32,33^ Lanthanum oxide coatings, when applied via spray coating methods and combined with fluorinated hyperbranched polyurethane (F-HPU), have been shown to impart self-cleaning properties onto glass while also producing robust coatings capable of withstanding harsh abrasion tests. Similarly, superhydrophobic cerium oxide coatings, applied to silane treated surfaces, have demonstrated improved thermal stability for engineering materials like copper, aluminum, and steel.

Furthermore, REOs are not confined to application in materials science; they also offer a wide range of uses in catalysis and biomedicine. In catalysis, REOs are utilized in water-resistant processes where hydrophobic environments are essential for preventing water deactivation and enhancing the adsorption of organic compounds.^34,35^ Fronzi et al. report density functional calculations that detail CeO_2_ exhibits hydrophobic contact angles of up to 112.53°, along with improved catalytic activity, by stabilization of oxygen vacancies.^36^ Likewise, in biomedicine, REOs are used to create hydrophobic coatings on medical devices and implants.^37,38^ These coatings reduce bacterial adhesion and can improve biocompatibility. REOs are also ideal for drug delivery systems and biosensors, as they offer improved stability and enable the controlled release of pharmaceutical compounds.^39,40^

The vast spectrum of applications of hydrophobic REO coatings ranging from medicine and catalysis to electronics and energy highlights the importance of fully harnessing their wettability for industrial advancements. This review provides an overview of the underlying mechanisms and synthesis methods influencing the wettability of REOs and specifically addressing the contrasting perspectives reported in the literature.

## 2 Unveiling the Hydrophobicity of REOs

### 2.1 Fundamental Properties

REOs are predominantly trivalent, with the 4f orbital being progressively filled across the lanthanide series. Despite differences in electronic configuration, their outermost electron structure (typically 6s^2^) imparts similar chemical behavior.^41^ REOs readily form M^3+^ ions, leading to basic oxides (RE_2_O_3_) and hydroxides. Their electropositive nature, high melting points, strong metallic bonds, and magnetic properties (due to unpaired 4f electrons) underpin their wide use in catalysis, magnetics, optics, and electronics.^22^ The structural diversity of REOs further complements their electronic uniformity, with RE_2_O_3_ phases adopting polymorphs such as A-type (hexagonal), B-type (monoclinic), and C-type (cubic), while some REEs (e.g., Ce) also stabilize in the tetravalent REO_2_ fluorite structure (Figure 2a).^5^ Additionally, REEs can be incorporated into complex oxide frameworks such as perovskites and double perovskites (Figure 2b), either at the A- or B-sites (Figure 2c), broadening their structural and functional landscape. This variability in coordination and symmetry plays a critical role in surface interactions and is central to ongoing debates regarding the intrinsic hydrophobicity of REO surfaces.

**Figure 2:**
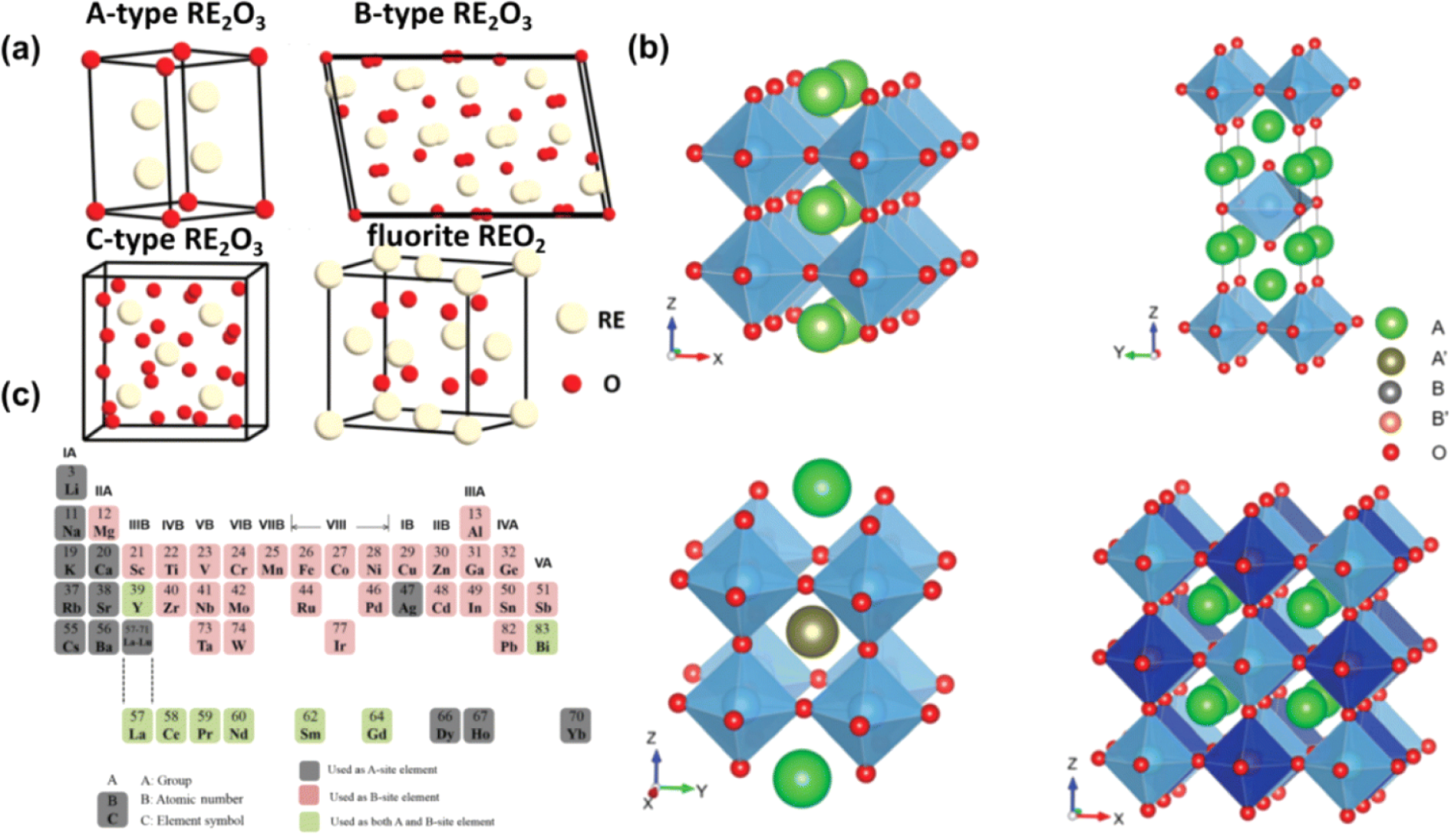
(a) Crystal structures of REOs, illustrating their inherent polymorphic diversity. (b) Representative perovskite and double perovskite configurations highlight the flexibility of REEs in octahedral frameworks. (c) Periodic table showing elements commonly used as A- and B-site cations in REE-based oxides. Reproduced with permission from ref 5. Copyright 2024 Royal Society of Chemistry.

In this trivalent state, the ionic metal–oxygen (M–O) bonds result in polar surfaces,^42^ implying inherently high surface energies typically associated with hydrophilicity. Moreover, shielding of the 4f orbitals limits outer electron interaction, further reinforcing expectations of hydrophilic behavior. However, while these bulk properties are consistent across the series, surface behavior does not always align.

### 2.2 Microstructure and Morphology

The microstructure of REOs encompasses a range of physical characteristics including particle size, shape, crystallinity, grain boundaries, porosity, and surface roughness. These attributes collectively dictate the macroscopic surface properties crucial for wettability. The specific synthesis method employed for REO nanoparticles or thin films profoundly influences these microstructural features, the availability of reactive sites, overall surface area, and the exposure of crystal facets. Consequently, understanding the microstructure of hydrophobic/hydrophilic surfaces is essential to estimating the wettability of REO surfaces.

Evidently, the lotus leaf (Nelumbo nucifera) has been used as the ideal model among researchers aiming to engineer advanced materials. Wherein the “lotus effect” originates from the lotus’ extremely low surface energy inherent of the hierarchical morphology present at the nanometer scale (Figure 3).^43^ The lotus effect refers to superhydrophobicity resulting from hierarchical micro/nano surface structures combined with low surface-energy waxes, leading to extreme water repellence. Its epidermal layer features regularly ordered, micrometer sized papillae that resemble spikes. These larger structures are further covered by an additional layer of nanometer sized wax tubules. This sophisticated multi scale roughness significantly minimizes the contact area between water droplets and the leaf surface. As depicted in Figure 3, the uniformly textured surface includes distinct valleys (70–100 nm) and flanges (3–10 μm). This intricate design, combined with the nanorod structures visible at higher magnifications, provides the optimal surface roughness necessary for the observed lotus effect. Ultimately, replicating such materials requires a deliberate combination of a low energy coating with targeted surface texturing.

**Figure 3:**
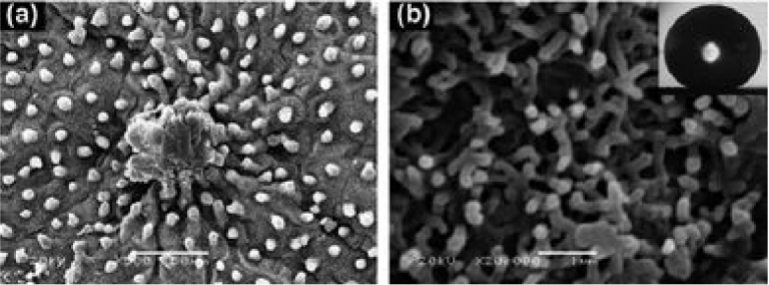
Tinted SEM image of a lotus leaf at low (a) (×500) and high (b) (×20,000) magnifications, with the inset of WCA value of about 162° on (b). Adapted with Permission from ref 43. Copyright 2011 Royal Society of Chemistry.

While the lotus leaf has served as a natural blueprint for designing superhydrophobic surfaces, translating such hierarchical structuring to REO thin films requires careful control of both surface energy and morphology. To explore how these factors have been experimentally realized, Table 1 presents a summary of representative REO coatings developed using various deposition techniques. The table outlines correlations between microstructure and wettability, revealing that high contact angles are observed across a diverse range of surface morphologies, from nanograined structures to irregular petal-like textures. Evidently, the presence of a particular morphology alone does not necessarily guarantee hydrophobic behavior. Indicating that growth environment and surface chemistry often play a more decisive role than microstructure alone.

**Table 1:**
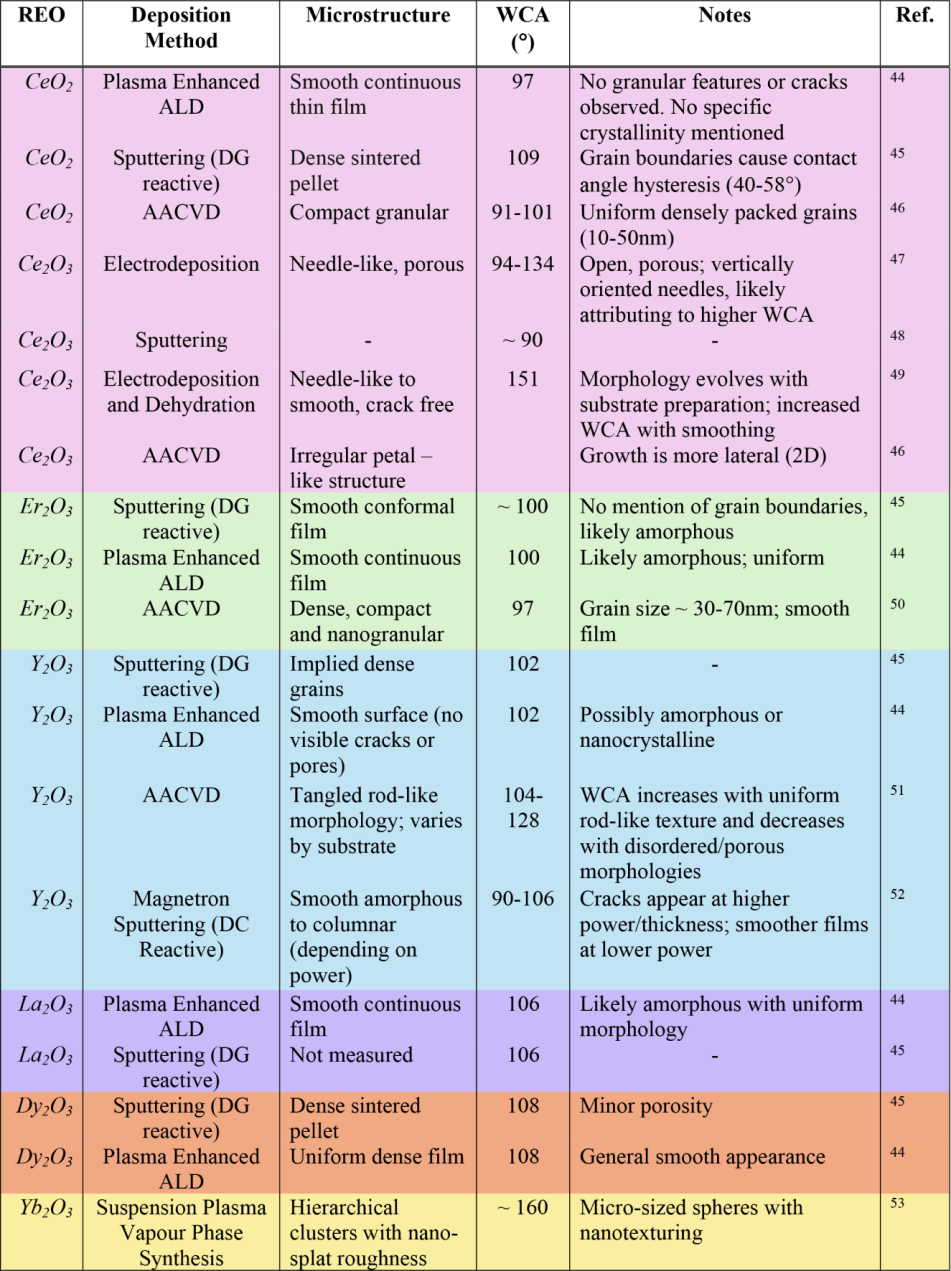
Reported Microstructures and Morphologies of Selected Rare-Earth Oxide (REO) Films from the Literature, along with Their Respective Deposition Methods and Measured Water Contact Angles (WCAs)^44–53^

### 2.3 The “Inherently Hydrophilic” Argument

Metal oxides are generally regarded as hydrophilic due to their polar M-O bonds and high surface energies. This is well established for oxides such as Al_2_O_3_ and TiO_2_,^54,55^ whose surfaces readily adsorb water and form hydroxyl groups that enhance hydrogen bonding.^56,57^ Similarly, REOs can display hydrophilic behavior when exposed to ambient conditions, where water adsorption and dissociation at defect sites leads to surface hydroxylation. This extrinsic hydrophilicity is supported by several lines of evidence: lanthanide-binding tags designed for aqueous environments, potentiometric studies of lanthanide­(III) complexes with organic acids (e.g., l-malic acid),^58^ and density functional theory (DFT) simulations that demonstrate hydration of lanthanide ions in solution.^59^ These findings indicate that while REOs may not be inherently hydrophilic, under typical environmental conditions, they can exhibit strong water affinity due to surface hydroxylation.

### 2.4 Evidence for Hydrophobic Behavior

In contrast, an established body of literature supports the view that REOs exhibit intrinsic hydrophobicity, generally attributed to their electronic structure.^34,45,48,60–63^ Azimi et al. proposed that REO surfaces inhibit hydrogen bonding due to a specific orientation of water molecules: only one hydrogen forms a weak bond with the oxide surface while the others engage more strongly with surrounding water, mimicking a hydrophobic hydration structure or anticlathrate configuration.^45^

The reduced reactivity of REOs is closely linked to the shielding of their 4f electrons by fully filled 5s^2^ 5p^6^ orbitals.^62,64^ For example, ceria’s (CeO_2_) hydrophobic nature has been explained as being due to the reduced availability of surface electrons for interaction with water. Zhu et al. demonstrated through first-principles calculations that water interacts differently with specific crystal facets of CeO_2_ (Figure 4).^64^ Whereby, water interacts differently with CeO_2_’s crystal planes: the (220) orientation has a lower adsorption energy (−0.750 eV) than the (111) surface (−0.639 eV), indicating that the absorption capacity of water molecules on the (111) surface is weaker, which has a higher WCA. Despite both orientations being hydrophobic, the degree of hydrogen bonding varies with the water molecule forming one hydrogen bond with (220) surface oxygen ion, whereas two hydrogen bonds with (111) oxygen ions, though still weaker than those seen on hydrophilic oxides. These findings indicate that even within a single REO, the degree of hydrophobicity can vary depending on crystal orientation, revealing a complex relationship between surface termination and water interaction.

**Figure 4:**
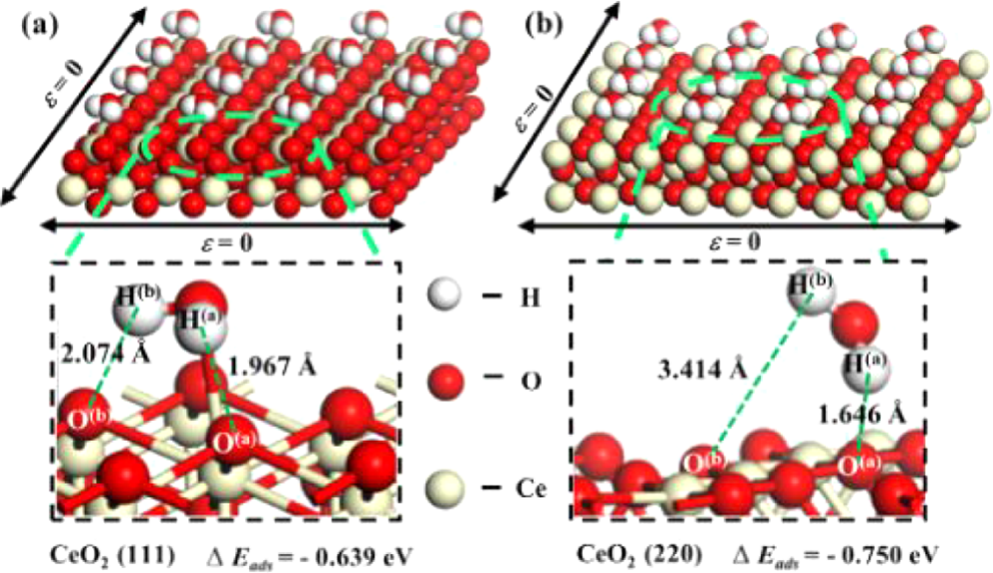
Optimized structures showing water molecule adsorption on CeO_2_ (a) (111) and (b) (220) surfaces. Adapted with permission from ref. 51. Copyright Elsevier.

Additionally, the strong oxophilicity of REOs results in chemically saturated oxide surfaces, where surface oxygen atoms are already tightly bound to metal cations. This reduces the likelihood of additional bonding with water molecules.^65,66^ Combined with the shielding of reactive electrons, this leads to weak surface water interactions, which is often manifested as water droplets beading up on the surface. In summary, while REOs possess many features that traditionally suggest hydrophilicity, their unique electronic and structural characteristics can also produce intrinsic hydrophobic behavior. Surface orientation, defect states, and environmental exposure all play key roles in determining their final wettability.

## 3 Emerging Insights: Deciphering the Hydrophobicity of REOs

### 3.1 The Role of Surface Structure and Termination

The wettability of rare earth oxides (REOs) is strongly influenced by their surface structure, particularly the ion-terminated surface (oxygen or rare earth). Unlike idealized models, REO surfaces exhibit different surface ion-terminated layers that significantly affect their interaction with water. For instance, in CeO_2_, the surface may terminate at either Ce^4+^ or O^2–^ layers. Oxygen or hydroxyl-terminated surfaces promote hydrogen bonding and thus hydrophilicity, while lanthanide cations-terminated (e.g., Ce^4+^) surfaces may hinder water adsorption due to differing electronic structures.^67^ Surface contamination, such as adsorbed hydrocarbons, can further obscure these terminations and modify wettability by masking the reactive surface.

Building on the observation that different crystal facets of REOs exhibit varying degrees of hydrophobicity, the specific surface structure, including atomic termination and defects, plays a decisive role in dictating water affinity. In CeO_2_, the (111) surface is the most stable, but whether it is Ce- or O-terminated significantly alters water adsorption behavior. O-terminated surfaces provide favorable sites for hydrogen bonding, enhancing hydrophilicity. In practice, REO surfaces also exhibit structural defects such as oxygen vacancies, step edges, and kinks.^68^ These features serve as active sites for water adsorption and dissociation.^69^ Therefore, the presence of oxygen vacancies enhances surface hydrophilicity by increasing the number of hydrogen bonding sites. The concentration of such defects depends on processing conditions, including synthesis methods, annealing temperatures, and atmospheric exposure.

Thus, surface terminated layers, structural defects, and adsorbates have a predominant influence on the overall wettability of REOs. A thorough understanding of these surface characteristics is essential for tailoring REO wettability in applications such as catalysis, coatings, and surface functionalization.

### 3.2 The Influence of Electronic Structure and Chemical Bonding

The electronic charge distribution across a REO surface, a critical factor when considering wettability, is governed by the nature of its M–O bonds. These bonds exhibit a spectrum of ionic and covalent character, which dictates the electrostatic interactions with polar water molecules. Comprehending the interplay between these bonding characters, influenced by factors such as electronegativity differences, ionic radii and charge states, and the specific surface orientation, is pivotal for designing REO surfaces with tailored wetting properties and functionalities.

While M–O bonds are often predominantly ionic due to the relatively high electronegativity of oxygen compared to many REEs, a degree of covalency is evident, particularly in oxides of higher valent rare earth metals (e.g., Ce^4+^).^70^ Recent advancements in understanding chemical bonding have highlighted the significance of charge-shift bonding (CSB) in metal oxides. The principles of CSB, where electron density “shifts” away from the atomic centers, suggest it could play a key role in the stability and properties of rare earth–oxygen (RE–O) bonds. The high electronegativity of oxygen, coupled with potential Pauli repulsion between oxygen and the rare earth metal centers, might indeed favor CSB contributions.^71^ Furthermore, the substantial ionic character typically associated with RE–O bonds aligns well with the importance of ionic resonance structures in CSB.

Drawing on the insights of Shaik et al., the formation of small, highly charged cations is known to promote CSB, especially with the presence of electronegative atoms (such as oxygen). Since REOs typically exist as trivalent cations, their relatively high charge density could contribute to CSB with oxygen anions or oxygen containing species at the REO surface.^71^ Likewise, the well-established oxophilic nature of REOs, which drives the formation of stable oxides, might be further enhanced by the charge-shift resonance energy associated with this type of bonding. Finally, the presence of f electrons in rare earth metals introduces an additional layer of complexity to their bonding. While these electrons are generally considered core like and not directly participating in bond formation, they indirectly influence the overall nuclear charge experienced by the valence electrons. In turn, impacting the ionic radii and electronegativity of the rare earth metal. Hence, modifying the ionic and covalent character of the RE–O bond and the surface’s interaction with water.

### 3.3 The Dynamic Nature of the REO–H_2_O Interface

The interaction of water with metal oxide surfaces is dictated by surface structure and defect density, which strongly influences whether molecular or dissociative adsorption occurs. In molecular adsorption, intact water molecules are adsorbed via weak van der Waals forces or hydrogen bonding, by the facilitation of surface oxygen atoms. Whereas, in dissociative adsorption, water molecules break down upon contact with the surface to give surface hydroxyl groups (−OH) and metal-hydroxide (M–OH) species.^72^

Hence each interaction pathway results in different wetting properties dependent on the surface morphology present. For example, molecular adsorption is more common on well ordered, stoichiometric surfaces with few defects as it involves weak interactions resulting in higher WCAs and hydrophobic behavior.^36,73^ While dissociative adsorption is favored at defect sites like oxygen vacancies, undercoordinated metal cations, and step edges. On reducible oxides like CeO_2_ and TiO_2_, oxygen vacancies serve as Lewis basic sites for hydrogen atoms, while cationic sites accommodate oxygen atoms, facilitating O–H bond cleavage.^74^ Surface hydroxylation enhances polarity and allows strong hydrogen bonding with additional water molecules, leading to lower WCAs and hydrophilic behavior. The mechanisms for molecular adsorption and dissociative adsorption can be seen in Figure 5 where water either remains intact or splits into OH^–^ and H^+^ fragments that bind to surface metal and oxygen atoms, respectively.

**Figure 5:**
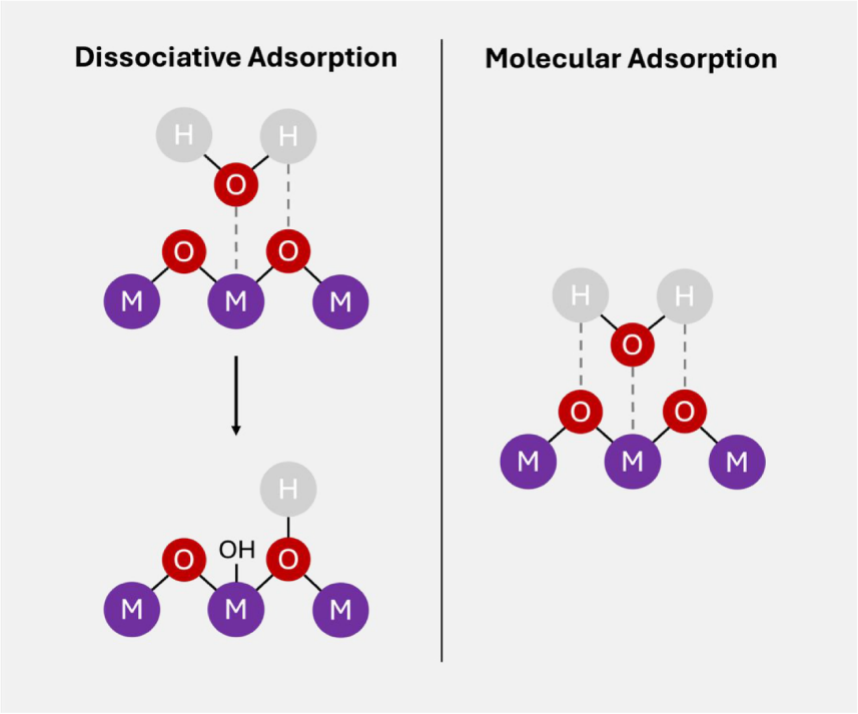
Schematic presenting the mechanism for molecular adsorption and dissociative adsorption.

It is important to note that while surface roughness or texturing can amplify existing wetting behavior, its effect depends on the intrinsic surface energy. On chemically hydrophilic REO surfaces, added roughness often enhances water spreading, whereas on low-energy surfaces contaminated with volatile organic compounds (VOCs), roughness can reinforce hydrophobicity or even induce superhydrophobicity. Thus, the presence of defects promotes dissociative adsorption and hydrophilicity, while their absence favors molecular adsorption and hydrophobicity. This provides a contrasting insight into the assumed hydrophobic behavior of REOs. This balance is fundamental to defining the wettability of REOs and can be tuned by controlling surface structure and defect density during synthesis or post treatment.

### 3.4 The Effect of Adsorbed Organic Species on Hydrophobicity

Recent literature reports discuss the mechanisms which govern the wettability of REOs and the significance of surface free energy. Oh et al. describe investigations into these mechanisms and the hydrophobicity of REOs derived from the adsorption of VOCs and rather than being intrinsically hydrophilic.^67^ Controlled hydrocarbon atmosphere environments and intrinsic surface chemistry analysis was conducted to reveal an increase in carbon content and C–C bonding over time, indicating hydrocarbon adsorption. This observation is consistent with recent findings from the deposition of ceria thin films via aerosol assisted chemical vapor deposition, wherein enhanced WCAs were observed upon exposure of the films to the atmosphere over time.^46^ Figure 6 presents enhancement in WCAs resulting from chemisorption and physisorption of hydrocarbons on the surface, as verified by XPS analysis, wherein chemisorption evidently enhanced further physisorption.^67^ These results led to the conclusion of the intrinsic hydrophilic nature of REOs and adapted hydrophobicity upon exposure to hydrocarbon environments.

**Figure 6:**
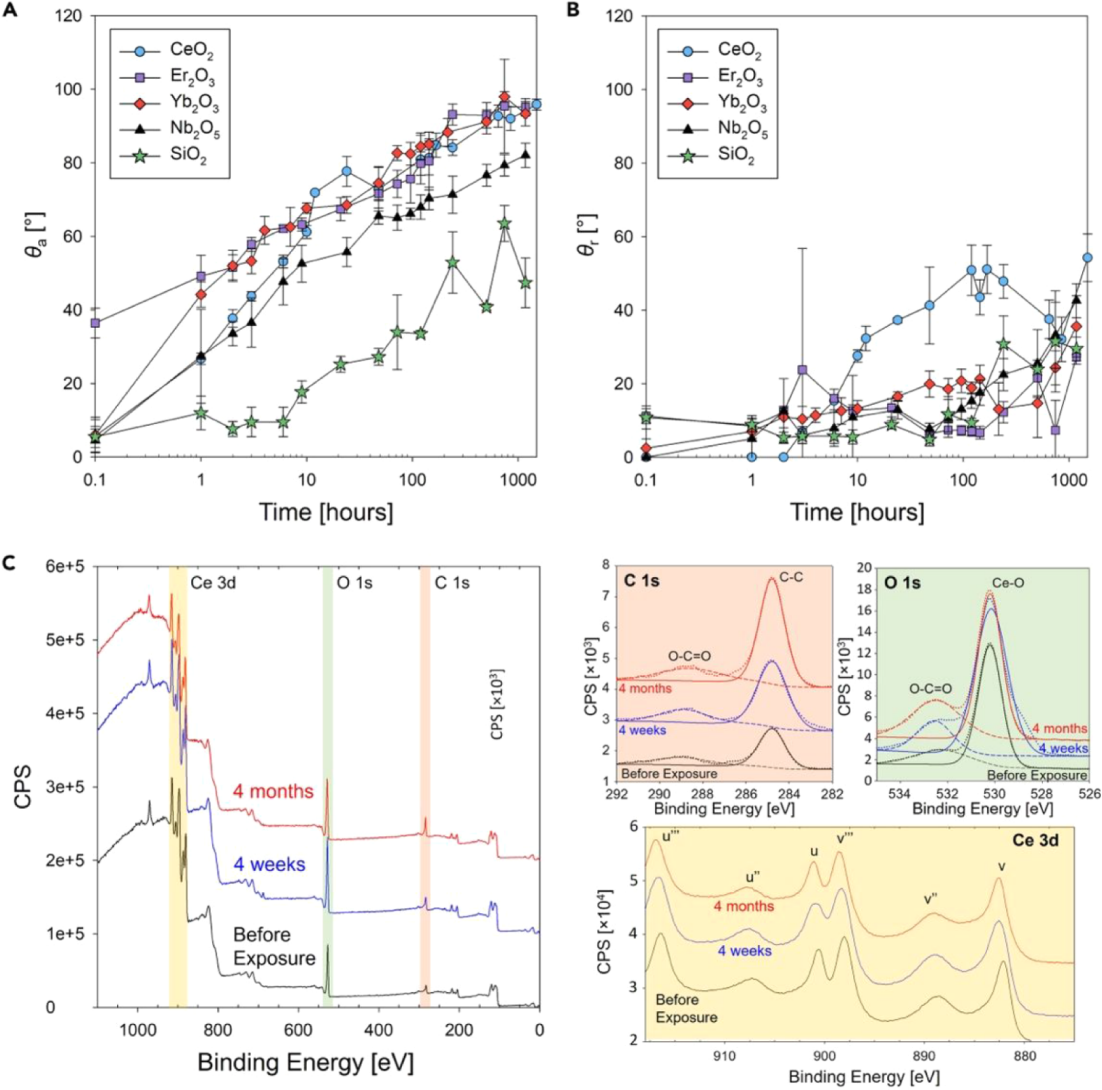
WCA measurements on REO coated Si wafers post extended exposure to the atmosphere. (a) advancing (θ_a_) and (b) receding (θ_r_) contact angles as a function of time on REO thin films (c) Broadband and high-resolution narrow-band XPS spectra for C 1s, O 1s, and Ce 3d. Reproduced from ref 67. © 2021 The Author(s). Published by Elsevier under a Creative Commons CC-BY license.

This data provides further evidence that pristine REO surfaces are intrinsically hydrophilic, and that pristine cleanliness can be achieved via plasma treatment of substrates. Oh et al. also reported that plasma cleaning introduces hydroxyl groups on the surface, which can enhance the hydrophilicity exhibited by REOs. However, consistency in the XPS spectra of the REOs and oxygen before and after plasma treatment suggested that hydroxylation was not the primary factor responsible for the initial hydrophilic character. Moreover, it was evident that throughout the adsorption process and despite the potential reduction of REOs, the chemical state of CeO_2_ remained remarkedly stable. The Ce 3d spectral region confirmed that the oxidation state of cerium (Ce^4+^) did not undergo significant change during 100 h of air exposure. Collectively, these observations indicate that the evolution of surface wettability was predominantly dictated by the adsorption of airborne contaminants rather than changes to the intrinsic chemical state of the REO surface.

Developing on this insight, studies conducted by Lundy et al. confirm the theory that REOs are intrinsically hydrophilic, with hydrophobicity arising upon exposure to external agents.^75^ Specifically, clean, pristine REO surfaces such as ceria exhibit low, hydrophilic WCAs, which has been consistently reported across the literature. Lundy et al. also attribute the development of hydrophobicity in REOs to the adsorption of VOCs on the surface. This adsorption process lowers the surface energy, thereby increasing the WCA. The study additionally emphasizes that the degree of surface hydroxylation had a direct correlation with contact angle hysteresis. The adsorption of nonane on ceria surfaces followed similar kinetics for both oxidation states, with surface coverage approaching approximately 0.65 at longer exposure times. In contrast, adsorption of species such as perfluorononane, resulted in markedly different wetting behaviors, underscoring the importance of specific oxide-adsorbate interactions.

In summary, growing evidence across the literature supports the emerging consensus that REOs are intrinsically hydrophilic, and that the commonly observed hydrophobicity in REOs arises from adsorption processes occurring at the surface.

## 4 Tailoring Wettability; Synthesis and Surface Modification Strategies

### 4.1 Influence of Synthesis Methods

As previously discussed, the surface morphology and molecular structure of REO surfaces play a critical role in determining whether they exhibit hydrophobic or hydrophilic behavior. Therefore, understanding and controlling synthetic routes to REO nanoparticles and thin films is essential for tailoring their wettability. In general, REO nanoparticles tend to exhibit hydrophilic behavior, making them suitable for biomedical applications. However, synthesis methods such as atomic layer deposition (ALD), hydrothermal processing, plasma spraying, and combustion can dramatically influence particle size, shape, surface roughness, and crystal facet exposure, which often results in surfaces with hydrophobic characteristics.

Recent studies using ALD to deposit REOs such as erbium oxide (Er_2_O_3_) and dysprosium oxide (Dy_2_O_3_), have demonstrated hydrophobic WCAs water contact angles (Figure 7) comparable to those achieved with yttrium oxide (Y_2_O_3_), lanthanum oxide (La_2_O_3_), and cerium oxide (CeO_2_).^44^ Interestingly, the hydrophobicity of these ALD deposited films decreased significantly after annealing at 500 °C, likely due to increased surface hydroxylation and moisture absorption. The drop in hydrophobicity attained upon annealing can be seen in Figure 7. Although there is a significant drop in WCA, the REOs remain marginally hydrophobic, and the initial WCA values still reflect marked hydrophobicity. Although the WCAs decrease upon exposure or treatment, they typically remain in the range of ∼60–80°, indicating that REO surfaces retain moderate hydrophobicity. These values suggest a shift away from strong hydrophobicity but remain well above the threshold of hydrophilic behavior. Except for La_2_O_3_, which exhibits a pronounced 80% reduction in WCA, dropping into the hydrophilic range. The underlying cause of this sharp decline is not discussed in the study, highlighting a gap in understanding regarding the surface behavior of La_2_O_3_.

**Figure 7:**
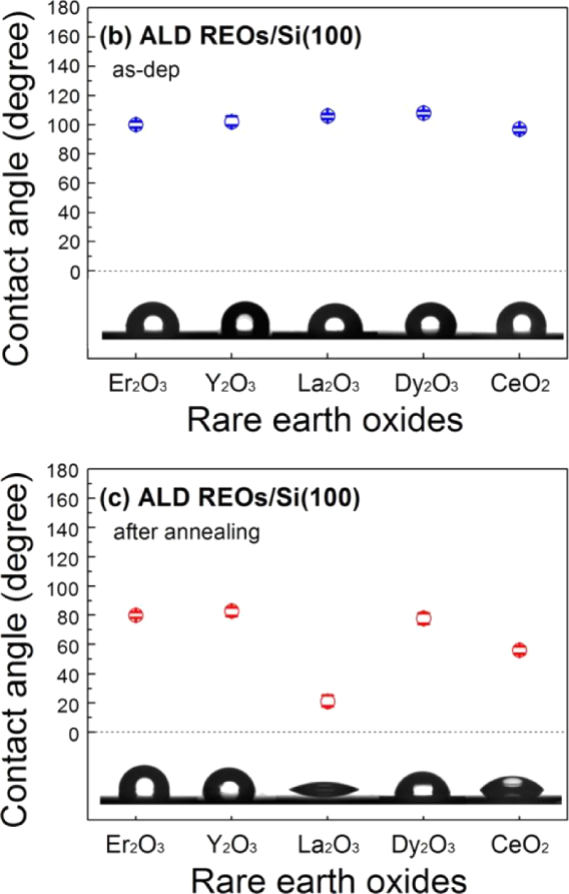
Water contact angles attained upon the deposition and annealing of lanthanide oxide films adapted from Oh et al. Adapted with permission from ref 44. Copyright 2015 American Chemical Society.

Notably, ALD was also used to conformally coat three-dimensional silicon nanowire arrays with Y_2_O_3_, yielding superhydrophobic surfaces with contact angles as high as 158°.

Borowiec et al. reported the synthesis of Er_2_O_3_ and Y_2_O_3_ thin films via chemical vapor deposition (CVD), achieving water contact angles of up to 128.3° (similar to those reported by Oh et al.).^50,51^ The Y_2_O_3_ films exhibited uniform rod-like morphologies and large crystallite sizes, with the most hydrophobic films showing fewer lattice defects and a preference for the cubic Y_2_O_3_ phase (Figure 8).^51^ Although annealing was not explicitly investigated, XPS analysis revealed that the most hydrophobic film had the lowest concentration of surface Y–OH groups and reduced hydrogen bonding, highlighting the key role of surface hydroxylation in modulating wettability. Importantly, the study also emphasized that the type of glass substrate onto which the REO film was deposited significantly influenced the resulting hydrophobicity. This was evidenced by the variation in surface morphologies and corresponding degrees of hydrophobicity observed across different substrates. These findings support the growing consensus that REOs are not intrinsically hydrophobic; rather, their wettability is dictated by external conditions and synthesis parameters.

**Figure 8:**
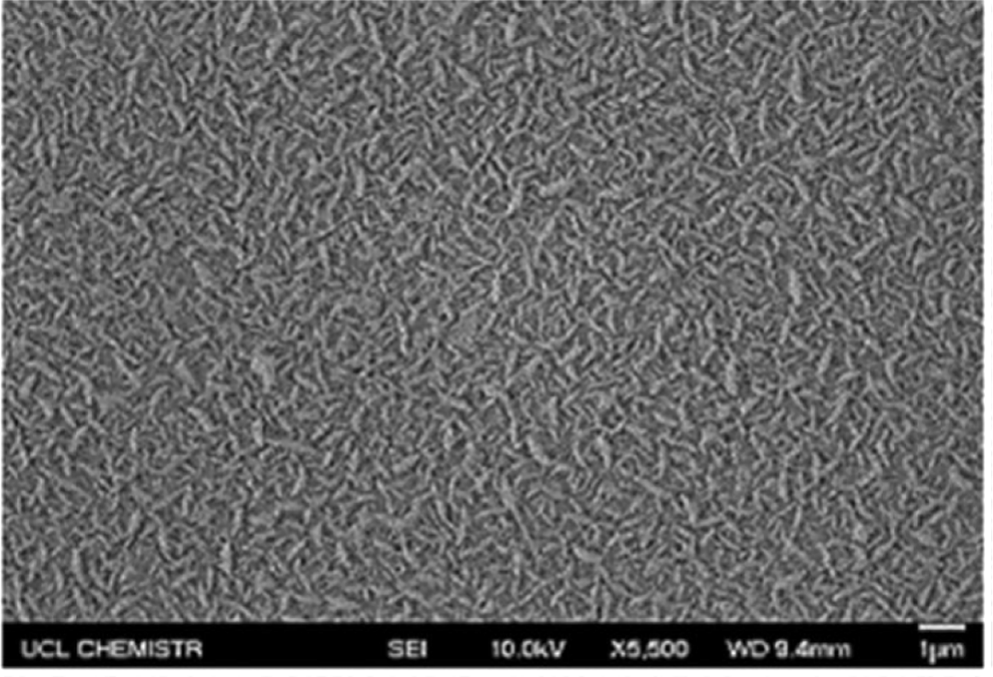
Morphology of yttrium oxide thin film, displaying rod-like structures of the most hydrophobic coating attained in the study. Adapted with permission from ref 51. Copyright 2024 Elsevier.

Further to this, the synthesis of pristine cerium oxide (CeO_2_) surfaces undoubtedly demonstrates hydrophilic behavior,^63^ where water molecules readily react with and sheet across the surface due to strong interactions with surface sites. This inherent affinity for water is strongly influenced by the synthesis method employed. Methods such as alkaline precipitation, hydrothermal processing, and sol–gel synthesis typically yield hydrophilic ceria due to their influence on particle size, surface area and defect structure.^76–78^ The importance of these factors on the wettability of REOs is imperative, as previously discussed. These wet chemical routes often produce nanostructured particles with high surface areas, exposing more active sites for water interaction, enhancing both molecular and dissociative adsorption. The aqueous environments used in these synthesis methods also favor hydroxylation, further increasing surface polarity and lowering WCAs.

Additionally, moderate levels of oxygen vacancies (common in ceria synthesized via sol–gel and precipitation) can trap and dissociate water, reinforcing surface hydroxylation and hydrophilic behavior.^78^ However, in some cases defects may induce hydrophobicity by actually facilitating water adsorption. In essence, the hydrophilic behavior observed in ceria may not be purely intrinsic but strongly influenced by synthesis conditions. These findings likely extend to other REOs with similar electronic structures, implying wettability is a function of processing parameters rather than an intrinsic property.

### 4.2 Surface Modifications

The use of inorganic materials and surface texturing to enhance the hydrophobicity of REO coatings has become an active area of research. As previously discussed, ALD is frequently employed to deposit REOs as hydrophobic or protective coatings.^44^ In parallel, efforts to induce superhydrophobicity have focused on increasing surface roughness using micro- and nanoscale structuring. For instance, high aspect ratio substrates like silicon nanowires are often used to enhance the hydrophobic performance of REOs by introducing more surface defects and active sites for hydroxylation. Such textures enhance wetting behavior and are key to fabricating stable water-repellent surfaces. In addition, laser ablation has emerged as a reliable technique for directly texturing REO ceramics such as ceria, increasing hydrophobicity.^45^ In these cases, the inherently low surface energy of certain REOs makes them promising candidates for achieving durable superhydrophobicity through surface modification.

Alongside inorganic modifications, organic functionalization of REOs is also prevalent in the literature. For example, functionalizing REO nanoparticles with organic ligands can improve their dispersibility in aqueous environments, effectively increasing their hydrophilicity in that context.^58^ Conversely, to assemble stable hydrophobic surfaces, researchers should employ organic molecules with low surface energy to tailor the interfacial chemistry of REOs. These functional coatings reduce surface energy and the adsorption of potential contaminants, thereby preserving long-term hydrophobicity.

## 5 Implications and Future Directions

### 5.1 Industrial Applications

The emerging understanding of the intrinsic hydrophilicity of REOs, and growth of hydrophobicity from environmental contaminants, reveals significant implications when considering their use across a range of industrial applications.

In catalysis, where reaction pathways are dictated by surface interactions, introduction of carbonaceous layers on the surface of the material could alter the active sites and hinder (hydrophilic) catalytic activity. Thus, the synthesis and maintenance of pristine REO surfaces is crucial for harnessing their full hydrophilic potential in catalysis. The inherent hydrophilic nature of pristine REOs can be advantageous in biomedical applications such as drug delivery and bioimaging, where good wettability and interaction with aqueous biological environments are desired. Surface modifications to further enhance this hydrophilicity or to create specific functionalities for targeted delivery will be an important avenue for future research. The ability to deliberately control surface wettability is also critical for advanced coating technologies, including applications from antifogging surfaces (which require sustained hydrophilicity) to self-cleaning materials, which rely on intrinsic hydrophobicity achieved through surface texturing and exposure of REO coatings to organic species.

### 5.2 Addressing Contrasting Perspectives

Evidently, there are many emerging contrasting perspectives on the wettability of REOs, highlighting their intrinsic hydrophilicity. The evidence strongly suggests that pristine REOs are inherently hydrophilic, and the observation of hydrophobic behavior often stems from the adsorption of airborne organic contaminants. This realization leads toward a reevaluation of past studies with investigations on rigorous surface cleaning and exposure to controlled environments. Understanding the kinetics and thermodynamics of VOC adsorption on different REO surfaces and under various conditions will be imperative for predicting their wettability.

### 5.3 Future Directions

Clearly there are several key areas that require further research to fully harness the full potential of REOs. Developing in situ techniques to characterize the wettability of REO surfaces under ultrahigh vacuum or controlled gas environments is essential to revealing their intrinsic properties without the influence of contaminants. Additionally, research into long-term strategies for maintaining pristine hydrophilic surfaces or stabilizing modified hydrophobic surfaces in real world conditions is critical. Advanced synthesis and surface modification techniques should be explored to achieve precise control over wettability patterns. DFT studies should also be continually employed to investigate the interaction of water, VOCs and organic contaminants with different REO surfaces. Enabling a deeper insight into the intrinsic properties of these metal oxides and the influence of surface terminations and defect sites on the adsorption mechanisms occurring.

### 5.4 Concluding Remarks

In conclusion, the hydrophobic nature of rare earth oxides is a complex phenomenon influenced by both their intrinsic surface chemistry and their interaction with the surrounding environment. Emerging evidence points toward an inherent hydrophilic nature that can be readily masked by the adsorption of volatile organic compounds. Acknowledging this emerging perspective is crucial for accurately interpreting existing literature and guiding future research. By developing robust methods for preparing clean REO surfaces and employing surface modification techniques, the full potential of REOs can be harnessed for a wide array of materials applications.
